# Astragalus polysaccharide prevents heart failure-induced cachexia by alleviating excessive adipose expenditure in white and brown adipose tissue

**DOI:** 10.1186/s12944-022-01770-3

**Published:** 2023-01-21

**Authors:** Dufang Ma, Tao Wu, Yiwei Qu, Jinlong Yang, Lu Cai, Xiao Li, Yong Wang

**Affiliations:** 1 Department of Cardiology, Shandong University of Traditional Chinese Medicine Affiliated Hospital, Shandong 250014 Jinan, China; 2 Shandong University of Traditional Chinese Medicine, Jinan, China

**Keywords:** Cardiac cachexia, Astragalus polysaccharide, Adipose expenditure, White adipose tissue, Brown adipose tissue

## Abstract

**Background:**

Astragalus polysaccharide (APS) is a key active ingredient isolated from Astragalus membranaceus that has been reported to be a potential treatment for obesity and diabetes by regulating lipid metabolism and adipogenesis, alleviating inflammation, and improving insulin resistance. However, whether APS regulates lipid metabolism in the context of cachexia remains unclear. Therefore, this study analysed the effects of APS on lipid metabolism and adipose expenditure in a heart failure (HF)-induced cardiac cachexia rat model.

**Methods:**

A salt-sensitive hypertension-induced cardiac cachexia rat model was used in the present study. Cardiac function was detected by echocardiography. The histological features and fat droplets in fat tissue and liver were observed by H&E staining and Oil O Red staining. Immunohistochemical staining, Western blotting and RT‒qPCR were used to detect markers of lipolysis and adipose browning in white adipose tissue (WAT) and thermogenesis in brown adipose tissue (BAT). Additionally, sympathetic nerve activity and inflammation in adipose tissue were detected.

**Results:**

Rats with HF exhibited decreased cardiac function and reduced adipose accumulation as well as adipocyte atrophy. In contrast, administration of APS not only improved cardiac function and increased adipose weight but also prevented adipose atrophy and FFA efflux in HF-induced cachexia. Moreover, APS inhibited HF-induced lipolysis and browning of white adipocytes since the expression levels of lipid droplet enzymes, including HSL and perilipin, and beige adipocyte markers, including UCP-1, Cd137 and Zic-1, were suppressed after administration of APS. In BAT, treatment with APS inhibited PKA-p38 MAPK signalling, and these effects were accompanied by decreased thermogenesis reflected by decreased expression of UCP-1, PPAR-γ and PGC-1α and reduced FFA β-oxidation in mitochondria reflected by decreased Cd36, Fatp-1 and Cpt1. Moreover, sympathetic nerve activity and interleukin-6 levels were abnormally elevated in HF rats, and astragalus polysaccharide could inhibit their activity.

**Conclusion:**

APS prevented lipolysis and adipose browning in WAT and decreased BAT thermogenesis. These effects may be related to suppressed sympathetic activity and inflammation. This study provides a potential approach to treat HF-induced cardiac cachexia.

**Supplementary Information:**

The online version contains supplementary material available at 10.1186/s12944-022-01770-3.

## Introduction

Cardiac cachexia is a dangerous condition caused by heart failure (HF) that can be defined as “unexplained rapid weight loss of more than 5% (or BMI < 20 kg/m^2^) within 12 months with concomitant conditions such as decreased muscle strength, fatigue, anorexia, low fat-free mass index and abnormal blood biomarkers (elevated C-reactive protein and/or elevated interleukin-6 (IL-6), Hb < 12 g/dL, or low serum albumin (< 3.2 g/dL))”; at least three of the above must exist at the same time [1]. Clinical studies have found that 10-39% of HF patients will eventually develop cachexia. [2]. Cachexia is more frequent in patients with advanced HF and is an independent predictor of mortality in HF [2]. However, to date, there is a lack of effective drugs against cachexia clinically.

Several clinical studies have shown that compared with noncachectic patients, patients with advanced HF cachexia had lower total body weight attributed mainly to more visible fat mass consumption compared with noncachectic patients, indicating that fat becomes the primary tissue consumed in cachexia rather than muscle [3, 4]. Moreover, wasting of fat mass, but not of lean mass, was predictive of adverse outcomes in advanced HF [4]. Loss of fat not only increased susceptibility to infection but also increased circulation of free fatty acids (FFAs) due to lipolysis of WAT, resulting in ectopic fat deposition as observed in atherosclerosis and hepatic steatosis, eventually leading to multi-organ dysfunction [5]. Therefore, maintaining normal metabolism of adipose tissue is considered as a valuable therapeutic measure to improve the prognostic status of cachexia patients.

Despite the clear clinical relevance, the fundamental pathophysiological mechanisms of fat wasting in cardiac cachexia have not been fully elucidated. A major contributing element of cardiac cachexia is the elevated lipolysis and adipose browning in WAT as well as increased thermogenesis in BAT [6]. It has been described that browning of WAT occurs under HF conditions, which is characterized by increased energy expenditure, adipose lipolysis and thermogenesis [7]. The activated sympathetic nervous system and increased inflammatory cytokines, such as IL-6, are important factors leading to adipose loss in HF [6, 8]. Increased catecholamines and inflammatory cytokines initiate triacylglycerol catabolism by activating lipolytic enzymes and promote the transcription of thermogenesis genes, which leads to excessive lipolytic activities in WAT and thermogenesis in BAT [6].

Astragalus membranaceus, widely known as Huangqi in China, which is suitable for the treatment of deficiency disorders [9]; moreover, it is a commonly used Chinese patent medicine for patients with chronic HF [10–12]. APS is a crucial active constituent separated from Astragalus membranaceus, and it has universal pharmacological properties in many life activities. To date, the pharmacological mechanism and action of APS have been studied in depth; recent pharmacological studies have demonstrated its advantages in controlling blood lipids and blood sugar, and it also plays a considerable role in fighting inflammation, oxidative stress, fibrosis, hepatitis and tumors [13]. Recent studies have shown that APS has a potential effect on obesity treatment and diabetes by regulating lipid metabolism and adipogenesis, alleviating inflammation and improving insulin resistance [14–16]. However, whether APS regulates lipid metabolism in the context of cachexia is still not clear.

In this paper, the effect of APS on lipid metabolism and adipose expenditure in a salt-sensitive hypertension-induced cardiac cachexia rat model was analysed. This study concluded that APS prevented lipolysis and adipose browning in WAT and decreased BAT thermogenesis. Moreover, mechanistic studies demonstrated that APS suppressed sympathetic activity and inflammation. This study provides a potential approach to treat HF-induced cardiac cachexia.

## Materials and methods

Six-week-old male Dahl salt-sensitive rats (Vital River, Beijing, China), weighing 140–180 g, were caged with free access to food and water. All rats were maintained at 20–22 ºC and 60–65% humidity with a 12-h light/dark cycle. The animal study was approved by the Experimental Animal Ethics Committee of Shandong University of Traditional Chinese Medicine (Licence No. 2021-30).

### Experimental groups

Dahl salt-sensitive rats were used to create cardiac cachexia models as described in a previous study [17]. 6 week old DS rats were prone to develop hypertension after 5 weeks of high-salt (8% NaCl) diet, and developed congestive heart failure after 12 weeks of high-salt (8% NaCl) diet; on the contrary, DS rats fed only low-salt (0.3% NaCl) diet as a control group did not show hypertension or heart failure. This model can clearly demonstrate the metabolic disorder and increased inflammation in rats during the modeling process, as well as the transition from compensatory left ventricular hypertrophy to congestive heart failure.

After 1 week of adaptive feeding, Dahl rats were given a high-salt diet (8% NaCl, *n* = 18) for 12 weeks, except for the control rats, which were fed a normal salt diet (0.3% NaCl, *n* = 6). After 12 weeks of modelling, they were randomized into three drug treatment groups: Model group, Low-dose group and High-dose group (*n* = 6) [17]. APS (Cat No. A7970) was purchased from Solarbio, Beijing, China, and the purity was ≥ 90.0%. The control group was still fed the usual salt diet, while all other three groups were fed a high-salt diet. Meanwhile, rats in the low-dose and high-dose groups were intragastrically administered a low dose (200 mg/kg) and high dose (800 mg/kg) of APS once a day for 6 weeks, and rats in the model group were intragastrically administered pure water [10, 18]. Rats in the control group and the two APS groups were fed in pairs to match the daily food intake of the model group.

### Arterial pressure and bodyweight

The systolic and diastolic blood pressures of the rats were measured before (12th week) and after (18th week) administration of APS by tail-cuff blood pressure multichannel system (IITC Life Science, California, USA). After warming up the tail of the rat, the measurement was performed. Each rat was measured five times, once every 5 min, and finally the average value was taken as the blood pressure value. Likewise, the body weight of the rats was also recorded regularly each week.

### Echocardiography

After the rats were anesthetized at the 12th and 18th weeks, indicators including left ventricular ejection fraction (EF) and left ventricular fractional shortening (FS) were measured using the M5 Veterinary Ultrasound system (Mindray, Shenzhen, China) at a heart rate of 380–420 beats/min, and cardiac function was compared before and after APS administration.

### Collection of blood samples and adipose tissue

At the end of the 18th week of feeding, each rat was anaesthetized with pentobarbital sodium (40 mg/kg, i.p.) and euthanized to collect fat, liver and other tissues. Blood samples collected from the inferior vena cava were centrifuged at 3000 rpm for 15 min at 4 °C to separate the serum and stored at − 80 °C for ELISA. Epididymal fat, inguinal fat and brown fat in the scapula were removed as quickly as possible on ice, recorded as a total fat weight and the ratio of fat weight to body weight was used as an index to assess fat mass. The epididymal fat and brown fat in the scapula were immediately stored in liquid nitrogen for subsequent experimental testing [19].

### In vitro glycerol measurements

Each group of epididymal fat was minced into 100 mg pieces and placed in KRBH buffer (Cat No. G0430, Solarbio, Beijing, China) supplemented with 10 µl isoproterenol. Samples were incubated at 37 °C for 60 min with gentle agitation. Subsequently, removed the medium and measured the level of glycerol released from the adipose tissue using a glycerol detection kit according to the kit’s instructions (Cat No. Ab133130, Abcam, Cambridge, United Kingdom).

### Haematoxylin and eosin (H&E) staining and oil red O staining

After embedding epididymal fat and brown fat with paraffin, cut into 4-µm thick sections and subsequently stained with HE and Oil Red O staining (Servicebio, Wuhan, China). Finally, tissue sections were viewed under a Nikon microscope (Nikon Eclipse E100, Nikon, Tokyo, Japan). Images were obtained from three random regions of each slice. The analysis was performed with a Nikon DS-U3 (Nikon, Tokyo, Japan).

### Immunohistochemical staining

The fixed adipose tissue was embedded in paraffin, cut into 8 μm thick sections, deaffinated with xylene and incubated with hydrogen peroxide (3%) for 25 min. Sections were blocked followed by the application of 3% BSA for 30 min. They were then incubated with anti-β3 adrenergic receptor (β3 AR) (1:200, Cat No. bs-10921R, Servicebio, Wuhan, China), anti-tyrosine hydroxylase (Th) (1:1000, Cat No. GB11181, Servicebio, Wuhan, China), anti-uncoupling protein-1 (UCP-1) (1:200, Cat No. GB112174, Servicebio, Wuhan, China), anti-hormone-sensitive lipase (HSL) (1:200, Cat No. 17333-1-AP, Servicebio, Wuhan, China), and anti-perilipin (1:500, Cat No. GB112022, Servicebio, Wuhan, China) primary antibodies overnight at 4 °C, washed and then incubated with goat anti-rabbit secondary antibody (1:200, Cat No. G1213-100UL, Servicebio, Wuhan, China) for 1 h at chamber temperature. Sections were counterstained with haematoxylin before being mounted. The stained slides were observed under a Nikon microscope (Nikon Eclipse E100, Nikon, Tokyo, Japan). Images collected from ten random fields per slice were finally analyzed using a Nikon DS-U3 (Nikon, Tokyo, Japan).

### Enzyme‑linked immunosorbent assay (ELISA)

The plasma levels of triglycerides (TGs) and FFAs and the levels of norepinephrine (NE) and IL-6 in adipose tissue were measured using high-sensitivity ELISA kits (TG (Cat No. JYM0046Ra), FFA (Cat No. JYM0208Ra), NE (Cat No. JYM0587Ra), IL-6 (Cat No. JYM0646Ra)); all these products were purchased from Colorfulgene Biological Technology, Wuhan, China. All steps of testing were performed according to product instructions attached to the product.

### Real-time quantitative PCR (qRT‒PCR)

Total RNA was extracted from the epididymal fat and brown fat in scapula using SparkZol Reagent (Cat No. AC0101, SparkJade, Jinan, China). Reverse transcription for cDNA used the SPARKscript II RT Plus Kit (Cat No. AG0304, SparkJade, Jinan, China). The total RNA quantification of the 20µL reverse transcription reaction system in this experiment was 1000 ng. The reaction conditions were as follows: Preincubation (1 cycle) 95 °C, 3 min; Amplification (40 cycles) 94 °C, 10 s, 60 °C, 25 s; and Dissociation (1 cycle) 95 °C,5s, 60 °C, 60 s; 97 °C, 15 s. Next, qRT‒PCR was performed using Light Cycler 480 SYBR Premix Ex Taq II (Roche, Basel, Switzerland). The mRNA expression levels were detected, and β-actin was used for normalization. The relative gene expression in the sample was calculated as 2^−ΔΔCT^. Experiments were performed in triplicate. The sequences of forward/reverse primers (synthesized by Tsingke, Beijing, China) are listed in Table 1.

**Table 1:** Primer sequences for real-time PCR

Gene	Sequence (5’–3’)
*β-actin*	Forward 5’-CCCATCTATGAGGGTTACGC’ Reverse 5’-TTTAATGTCACGCACGATTTC’
*Hsl*	Forward 5’-GCCAGCCACAACCTAGCAGAAC’ Reverse 5’-CATCGCCCTCAAAGAAGAGCACTC’
*Peripilin*	Forward 5’-GACGCTCCGTTCTTCAGAGAATCAC’ Reverse 5’-GTGCTGAGACTGATGGAAGGTGTG’
*Fas*	Forward 5’-GCTGTGGATCATGGCTGTCCTG’ Reverse 5’-TTCACGAACGCTCCTCTTCAACTC’
*Cd137*	Forward 5’-TTCTCTGGTTCTCTGTGCCCAAATG’ Reverse 5’-CCTCCTCCTCCTTCTTCTTCCTCTG’
*Zic-1*	Forward 5’-CCCAGAGCAGAGCAACCACATC Reverse 5’- CAGGAAACGGGCAGGGAAAAGG
*Lpl*	Forward 5’-AGACTCGCTCTCAGATGCCCTAC’ Reverse 5’-TCACTTTCAGCCACTGTGCCATAC’
*Cd36*	Forward 5’-TTCTTCCAGCCAACGCCTTTGC’ Reverse 5’-CTTCTTTGCACTTGCCAATGTCCAG’
*Fatp-1*	Forward 5’-GGCTGTGTATGGAGTGGCTGTG’ Reverse 5’-AGGAAGATGGGCTGGGCATAGG’
*Ppary*	Forward 5’-TCTGTGGACCTCTCTGTGATGGATG’ Reverse 5’-GTCAGCTCTTGTGAACGGGATGTC’
*Pgc-1α*	Forward 5’-ACCGCACACATCGCAATTCTCC’ Reverse 5’-AGACTCCCGCTTCTCATACTCTCTG’
*Cpt1*	Forward 5’-GAGCCACGAAGCCCTCAAACAG’ Reverse 5’-AAATCACACCCACCACCACGATAAG’
*Ucp-1*	Forward 5’-GCACCCGACCCAGAAAGACATC’ Reverse 5’-TCAGTTCACCTCCAGCACCTCAG’

### Western blot analysis

Protein was extracted from the interscapular BAT using the Minute™ Animal Adipose Tissue Protein Extraction Kit (Invent, Minnesota, USA). Adipose tissue homogenate was extracted using ice-cold RIPA buffer, and protein concentrations were determined using an enhanced bicinchoninic acid (BCA) protein assay kit (Cwbio, Jiangsu, China). The protein concentration determined by the BCA kit was finally prepared into a normalized protein system with a concentration of 2 µg/µL. Then, 20 µg of protein from each group was separated by sodium dodecyl sulfate‒polyacrylamide gel electrophoresis (SDS‒PAGE) and transferred onto polyvinylidene fluoride (PVDF) membranes (Millipore, Massachusetts, USA) for blotting. After incubation with 5% nonfat milk in TBST for 1 h at room temperature, the membranes were incubatedwith primary antibodies overnight for 8 h at 4 °C against glyceraldehyde 3-phosphate dehydrogenase (GAPDH) (1:2000, Cat No. ab181602, Abcam, Cambridge, United Kingdom), protein kinase A (PKA) (1:1000, Cat No. ab32390, Abcam, Cambridge, United Kingdom), phospho-PKA (p-PKA) (1:500, Cat No. Bs-3725r, Bioss, Beijing, China), p38 mitogen-activated protein kinase (p38 MAPK) (1:1000, ab31828, Abcam, Cambridge, United Kingdom), phospho-p38 MAPK (p-p38 MAPK) (1:500, Cat No. Bs-0636r, Bioss, Beijing, China), peroxisome proliferator-activated receptor γ coactivator 1α (PGC-1α) (1:2000, Cat No. Bs-7535r, Bioss, Beijing, China), peroxisome proliferator-activated receptor gamma (PPAR-γ) (1:2000, Cat No. Bsm-33,436 m, Bioss, Beijing, China) and UCP-1 (1:2000, Cat No. Bs-1925r, Bioss, Beijing, China). After using the TBST to wash the membrane five times (6 min each), the blots were incubated with the secondary antibody of the corresponding species for 1 h. After washing with TBST, the protein bands were visualized by chemiluminescence using the Sensitivity ECL Chemiluminescence Detection Kit (Proteintech, Wuhan, China). Finally, FluorChem Q 3.4 (ProteinSimple, California, USA) was used to measure the band intensities.

### Statistical analysis

The obtained data were statistically analyzed by SPSS 26.0 software (IBM, New York, USA). Data collected during the study are presented as the mean ± SD and were analysed by the single-factor analysis of variance method (ANOVA), followed by Dunnett’s t test for group comparisons. *P* < 0.05 was defined as statistically significant.

**Fig. 1:**
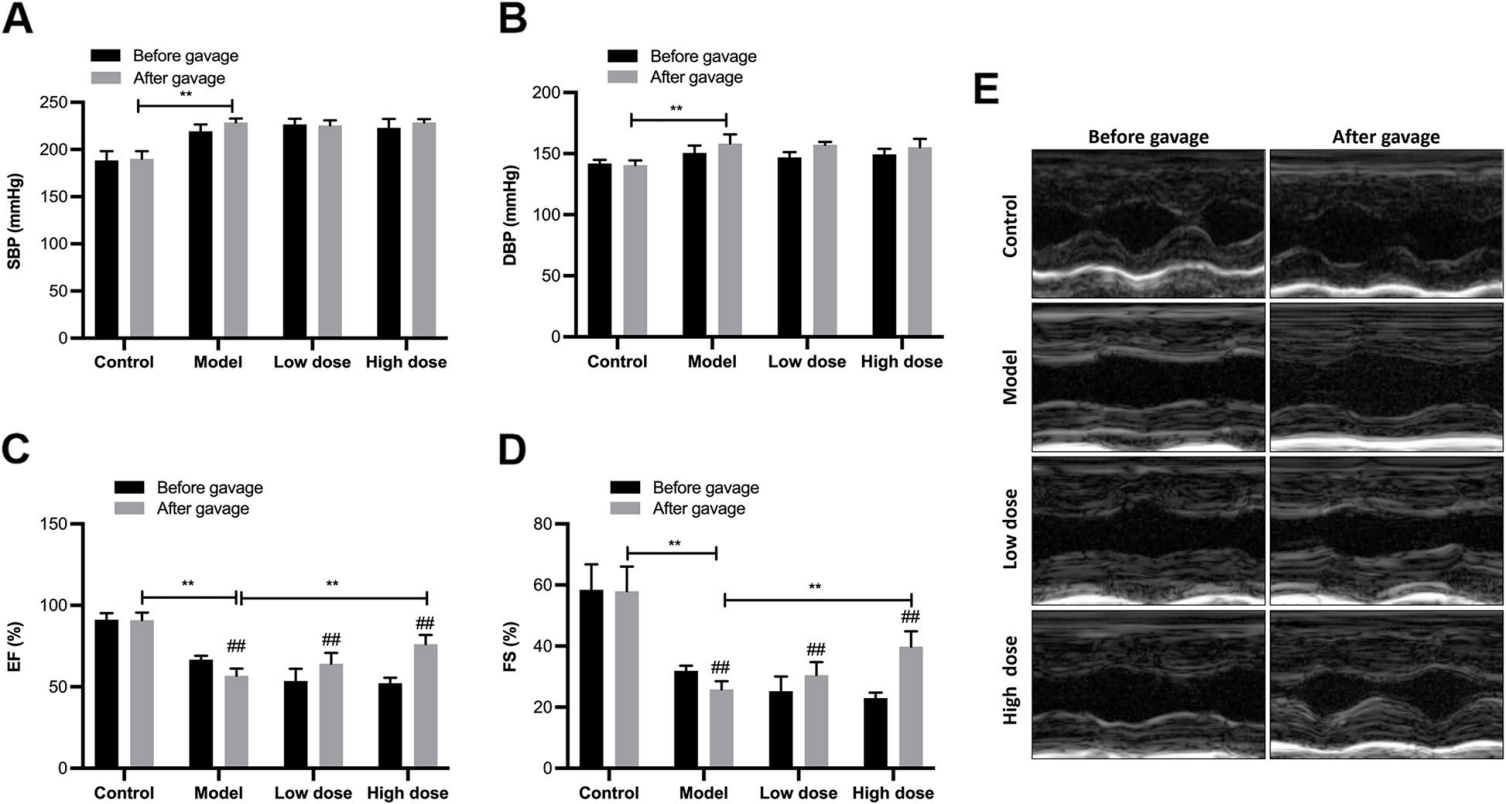
Effects of APS on blood pressure and cardiac function. **A** Systolic blood pressure (SBP), **B** Diastolic blood pressure (DBP), **C** Left ventricular ejection fraction (EF), **D** Left ventricular fractional shortening (FS), **E** Representative images of echocardiography. *P < 0.05, **P < 0.01. ^#^*P* < 0.05, g^##^*P* < 0.01, before gavage vs. after gavage, *n* = 6

## Results

### APS improved cardiac function in HF

As shown in Fig. 1, at the end of the 18th week, the blood pressure in the heart failure group was significantly higher compared with that of the control group (*P* < 0.01; Fig. 1A,B). APS had no impact on blood pressure. The cardiac function reflected by EF and FS showed that the rats in the model group had worse cardiac function (*P* < 0.01; Fig. 1C,D,E), while administration of a high dose of APS elevated EF and FS (*P* < 0.01), indicating that APS improved cardiac function in hypertension-induced HF. Echocardiography was performed after 18 weeks of feeding and the M-mode echocardiographic findings are shown in Table 2.

**Table 2:** Echocardiographic parameters in all groups at 18 weeks of age

	Control	Model	Low dose	High dose
LVPWd (cm)	0.17 ± 0.03	0.23 ± 0.02^a^	0.22 ± 0.02^a^	0.21 ± 0.01
LVIDd (cm)	0.50 ± 0.12	0.64 ± 0.04	0.57 ± 0.10	0.54 ± 0.06
LVIDs (cm)	0.47 ± 0.04	0.45 ± 0.04	0.44 ± 0.06	0.45 ± 0.07

*LVPWd* Left Ventricular Posterior Wall Dimensions, *LVDd* Left ventricular end-diastolic dimension, *LVDs* Left ventricular endsystolic dimension

Values are mean ± SD. ^a^*P* < 0.05 vs. Control group. *n* = 6 per group

**Fig. 2:**
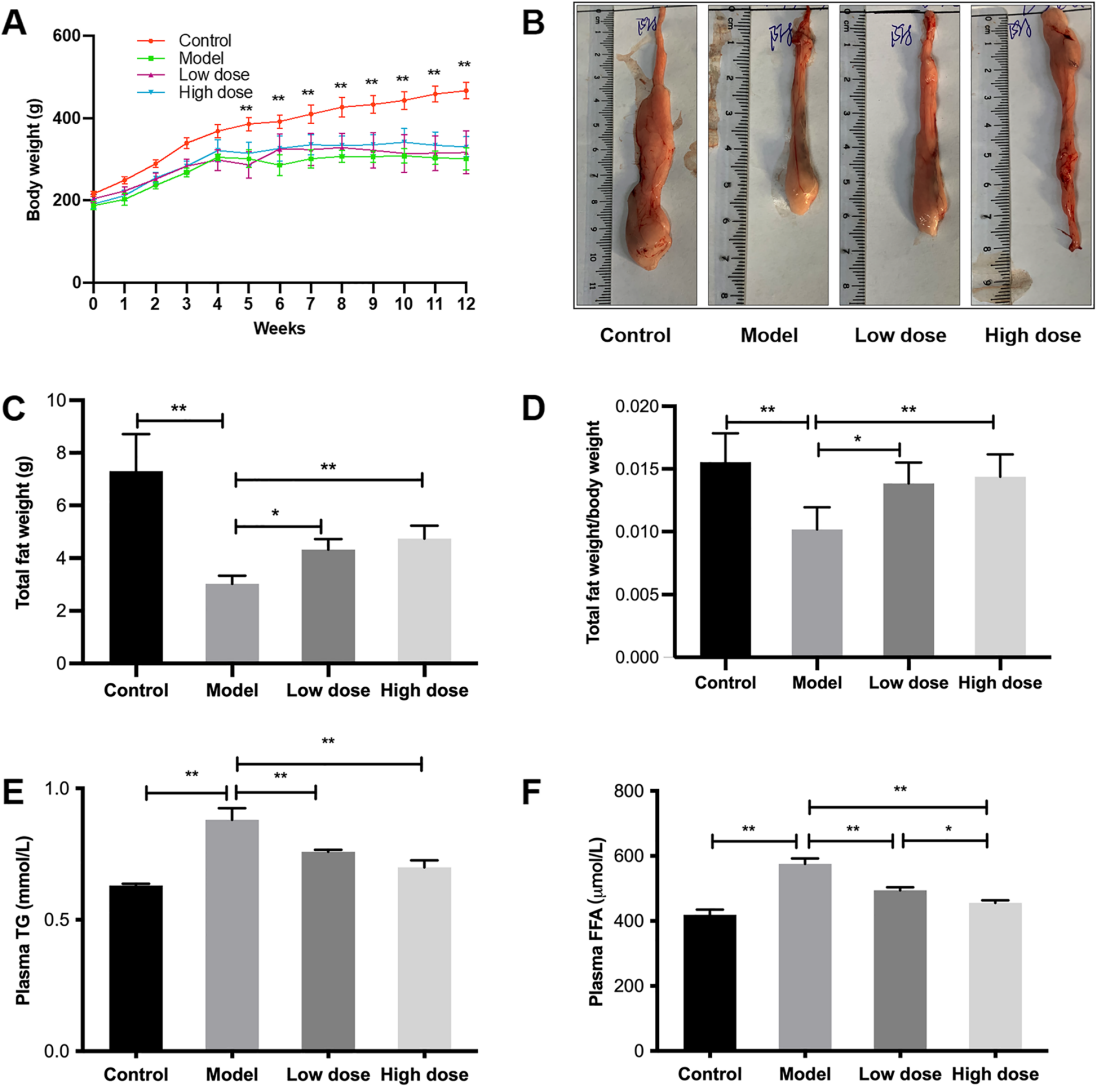
APS treatments prevent fat wasting in HF-induced cachexia. **A** Changes in body weight through the duration of the 12-week study, **B** Epididymal fat volume, **C** Total body weight, **D** Ratio of total fat weight and body weight, **E** Levels of plasma TG, **F** Levels of plasma FFA. *P < 0.05, **P < 0.01, *n* = 6

### APS treatments prevent fat wasting in HF-induced cachexia

At the end of the 18th week, the model group exhibited continuous weight loss throughout the duration of the study compared with the control group (*P* < 0.01; Fig. 2A). The administration of low- and high-dose APS alleviated weight loss, although no significant difference. Fat mass and the ratio of fat mass and body weight also significantly decreased compared with rats without HF (*P* < 0.01; Fig. 2B-D). Both high and low doses of APS prevented fat loss in HF rats (*P* < 0.01, *P* < 0.05; Fig. 2B-D). At the same time, the levels of TG and FFA in plasma were detected to indirectly reflect the lipid storage of fat, and it was found that the levels of TG and FFA in the model group increased (*P* < 0.01; Fig. 2E,F), while administration of APS decreased both the TG and FFA levels (*P* < 0.01); moreover, a high dose of APS decreased plasma FFA levels more significantly than a low dose of APS (*P* < 0.05).

**Fig. 3:**
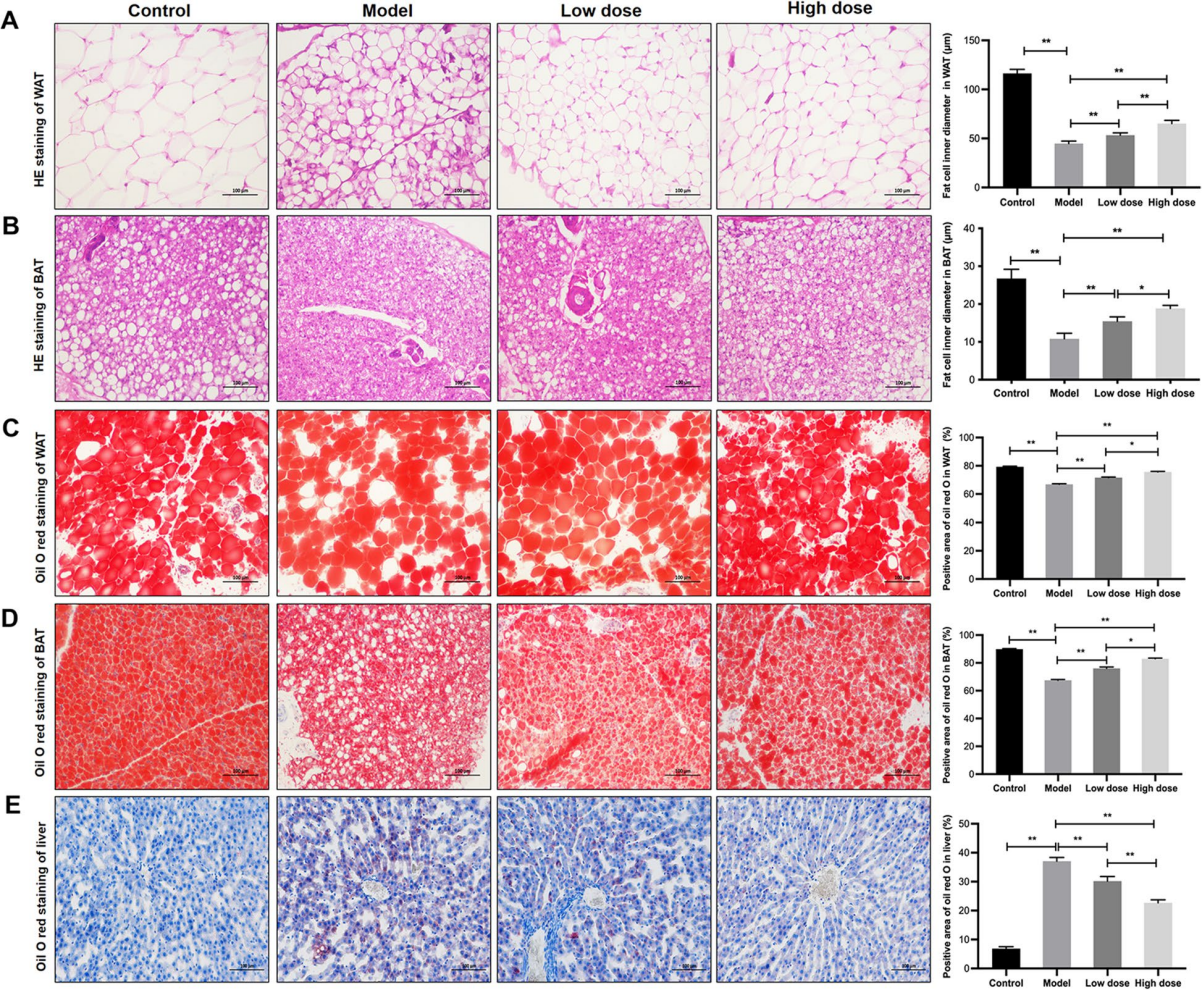
APS prevents adipose tissue atrophy and FFA efflux into the liver in HF-induced cachexia. **A** HE staining and fat cell diameter of WAT, **B** HE staining and fat cell diameter of BAT, **C** Oil red O staining and positive area of WAT, **D** Oil red O staining and positive area of BAT, **E** Oil red O staining and positive area of liver tissue. Scale bar = 100 μm. *P < 0.05, **P < 0.01, *n* = 6

### APS prevents adipocyte atrophy and ectopic lipid deposition in the liver in HF-induced cachexia

Histology and fat droplets in rat adipose tissue and liver were observed. Compared with rats in the control group, H&E staining of epididymal fat and brown fat in scapula showed smaller adipocytes in rats with HF (*P* < 0.01; Fig. 3A,B). Treatment with APS alleviated adipocyte atrophy, with high-dose APS showing a more significant effect (*P* < 0.01). Consistently, according to the results of Oil red O staining, it was found that the fat accumulation of epididymal fat and BAT of rats in the model group decreased, while ectopic fat accumulation was elevated in liver tissue compared with the rats without HF (*P* < 0.01; Fig. 3C-E). Both low and high doses of APS treatment increased fat accumulation in both white and BAT after 6 weeks of treatment and alleviated FFA influx into the liver (*P* < 0.01, *P* < 0.05). These results indicated that APS prevented adipocyte atrophy and FFA efflux caused by cachexia.

**Fig. 4:**
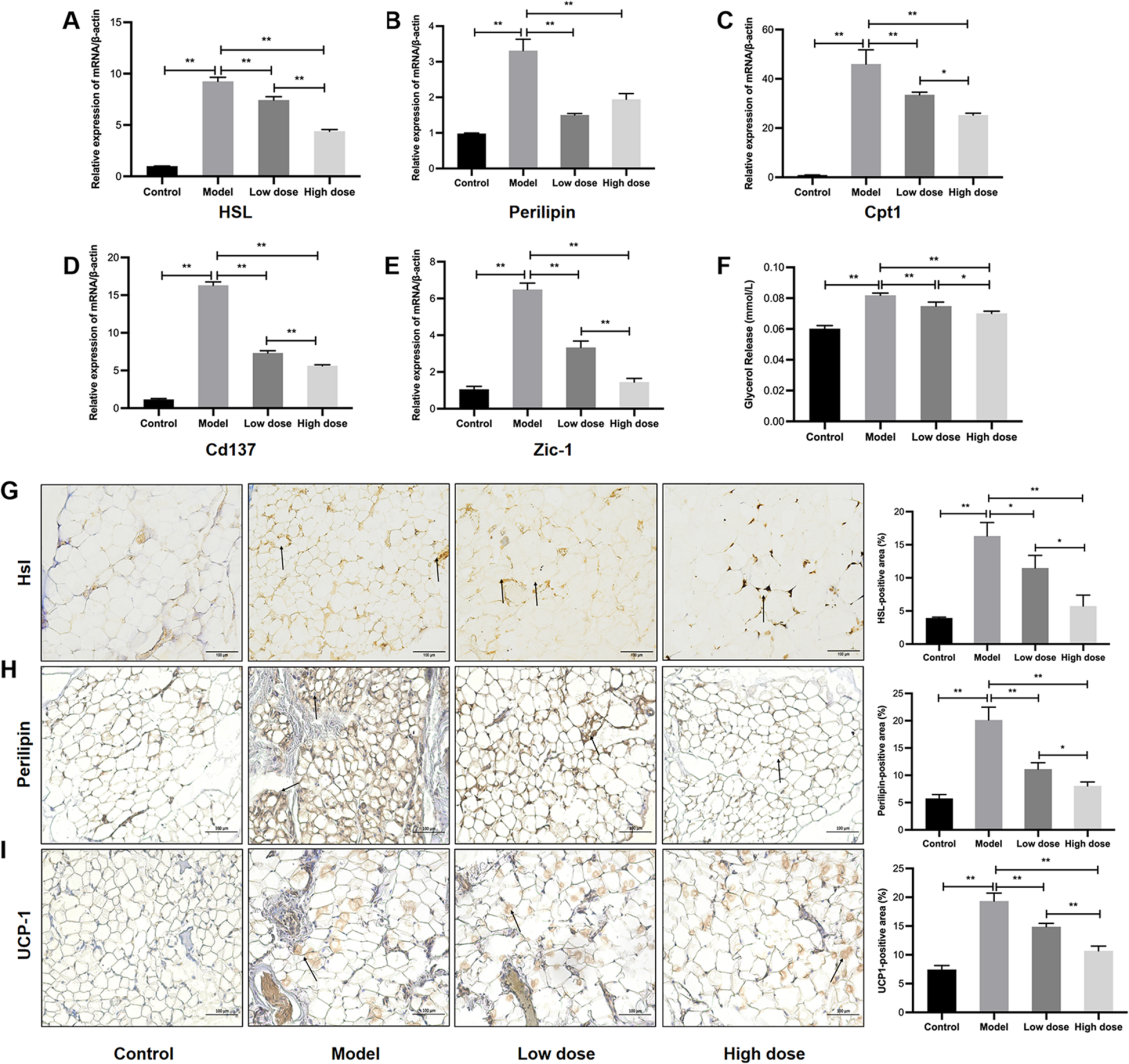
APS prevented lipolysis and lipid consumption in WAT. **A**, **B** mRNA expression of lipid droplet proteins, including *HSL* and *perilipin*. **C** mRNA expression of the fatty acid β-oxidation gene *Cpt1*. **D**, **E** mRNA expression of browning WAT markers, including *Cd137 and Zic-1*. **F** Glycerol release levels in each group after treatment with isoproterenol. **G**-**I** Immunostaining and positive areas of HSL, perilipin and UCP-1. Scale bar = 100 μm. *P < 0.05, **P < 0.01, *n* = 6

### APS prevented lipolysis and lipid consumption in WAT in HF-induced cachexia

NE released from sympathetic nerve terminals increases cellular concentrations of cyclic adenosine monophosphate (cAMP) by binding to β-adrenoreceptors (β-ARs) and activating adenylate cyclase (AC). PKA is activated by increased cAMP concentrations, which in turn phosphorylate lipid droplets, including HSL and perilipin. Eventually, TGs stored in lipid droplets are hydrolyzed into free fatty acids and released [20–22]. This study assessed whether APS inhibits the hydrolysis of TGs and the release of FFAs in WAT. According to the present study, lipolysis is activated in HF-induced cachexia, which is characterized by increased release of glycerol after administration of isoproterenol and elevated mRNA and protein levels of HSL and perilipin, whereas APS may inhibit lipolysis, which is characterized by the release of glycerol from adipose tissue in the APS intervention group being obviously less than that of the model group (*P* < 0.01; Fig. 4 A,B,F,G,H).

Similarly, the expression levels of the FFA β-oxidation gene carnitine palmitoyltransferase (*Cpt1)* and browning of WAT markers, including *Cd137, Zic-1* and UCP-1, were increased compared with rats without cachexia in the control group (*P* < 0.01; Fig. 4C-E,I) [23]. These results indicated that elevated lipid consumption was caused by increased FFA β-oxidation and WAT browning. Administration of APS decreased the mRNA expression levels of *Hsl, Perilipin, Cpt1, Cd137* and *Zic-1* and the protein expression levels of HSL, perilipin and UCP-1 (*P* < 0.05, *P* < 0.01), with a high dose of APS exhibiting significant effects. These results indicated that APS inhibited lipid consumption in WAT by suppressing the lipolytic pathway and decreasing fatty acid β-oxidation and WAT browning.

**Fig. 5:**
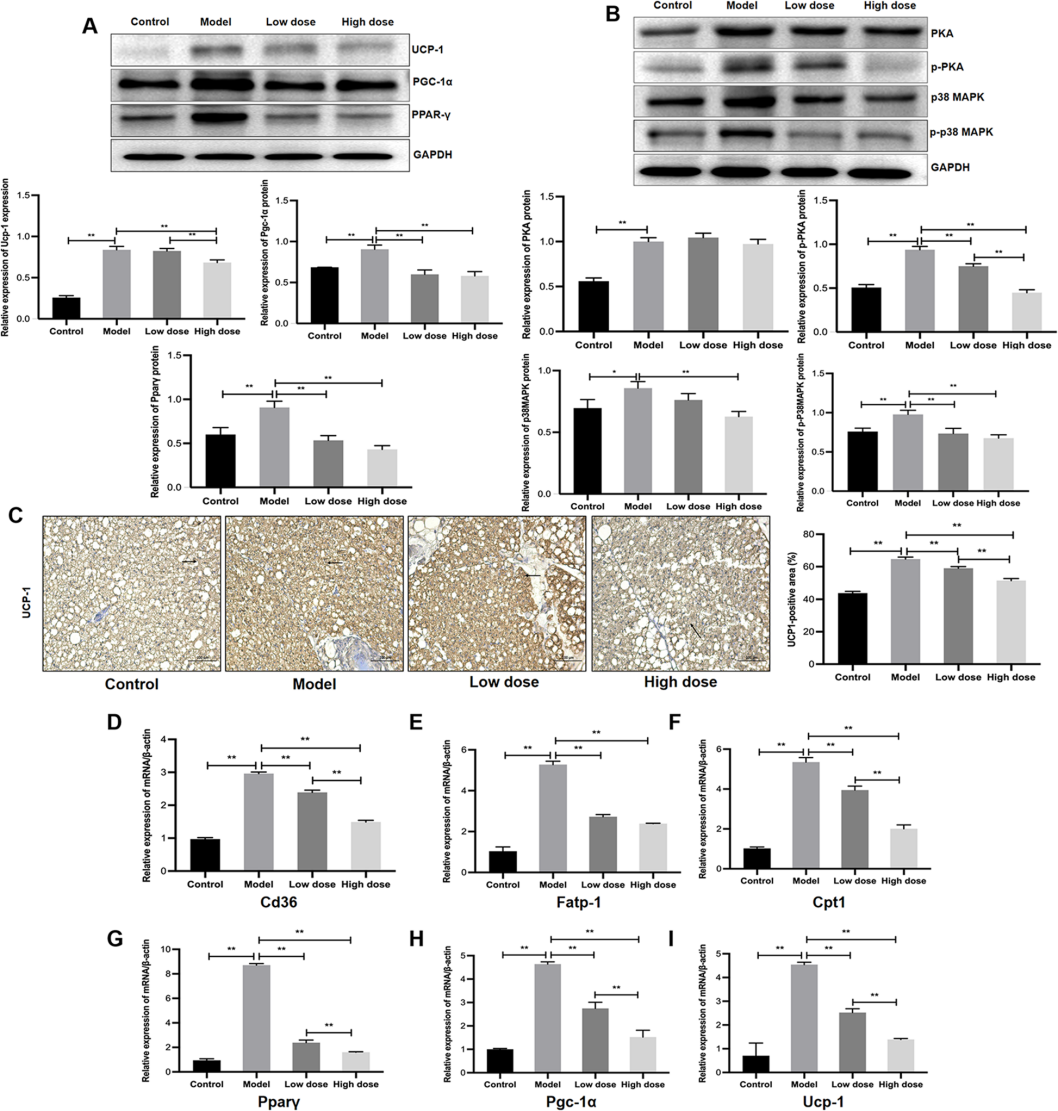
APS prevented thermogenesis in BAT. **A** Protein expression of PPAR-γ, PGC-1α and UCP-1 in BAT. **B** Protein expression of PKA, p-PKA, p38 MAPK and p-p38 MAPK in BAT. **C** Immunostaining and positive area of HSL in BAT. **D**-**F** mRNA expression of the transport proteins *Cd36*, *Fatp* and *Cpt-1*. **G**-**I** mRNA expression levels of PGC-1α, PPAR-γ and UCP-1. Scale bar = 100 μm. *P < 0.05, **P < 0.01, *n* = 6

### APS prevented PKA-induced thermogenesis in BAT in HF-induced cachexia

In brown adipocytes, the expression of UCP1 and other thermogenic genes is elevated by activated PKA signaling through the p38 MAPK cascade, which enhances thermogenesis in brown adipocytes [21, 22]. This study detected thermogenic capability in brown adipocytes. Compared with rats without cachexia, PKA-p38 MAPK signalling was activated in the model group, and the protein expression levels of PKA, p-PKA, p38 MAPK and p-p38 MAPK were enhanced (*P* < 0.05, *P* < 0.01; Fig. 5B). Meanwhile, with the increase in p38 MAPK activation seen in brown adipocytes, the mRNA and protein expression levels of key thermogenesis factors, including PPAR-γ, PGC-1α and UCP-1, were increased (*P* < 0.01; Fig. 5A,C,G-I) [24]. These results indicated that thermogenesis in BAT was increased in HF-induced cachexia. Treatment with APS, especially with a high dose of APS, prevented PKA-p38 MAPK signalling and inhibited the mRNA/protein expression of PGC-1α, PPAR-γ and UCP-1 (*P < 0.01*). These outcomes suggested that APS prevents PKA-p38MAPK signalling-mediated thermogenesis in brown adipocytes.

FFAs derived from white adipocyte lipolysis are taken up to brown adipocytes by transport proteins such as fatty acid transport protein (FATP) and CD36 and then transported to mitochondria via CPT-1 to serve as fuel for energy production [21]. HF-induced cachexia elevated the mRNA expression levels of *Cd36, Fatp-1* and *Cpt-1* (*P* < 0.01; Fig. 5D-F). Administration of a high dose of APS inhibited the mRNA expression of the three transport proteins (*P* < 0.01), with high-dose APS having more significant effects on *Cd36* and *Cpt-1* than low-dose APS (*P* < 0.01). This result indicated that APS suppressed FFA utilization and decreased thermogenesis in brown adipocytes.

**Fig. 6:**
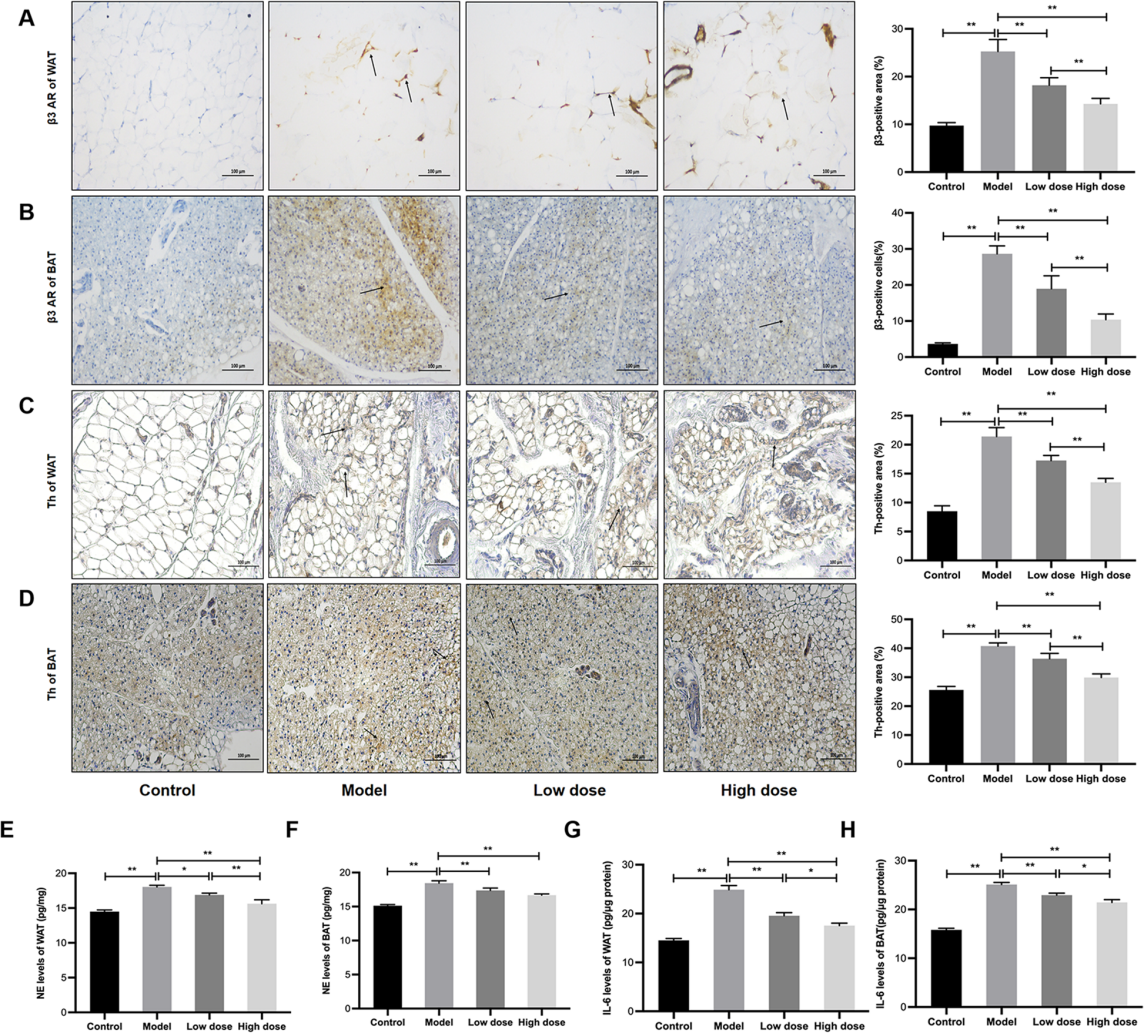
APS inhibited sympathetic activation and IL-6 in HF-induced cachexia. **A**, **B** Immunostaining and positive areas of β3 adrenergic receptor (β3 AR) in WAT and BAT. **C**, **D** Immunostaining and positive areas of tyrosine hydroxylase (Th) in WAT and BAT. **E**, **F** NE levels in WAT and BAT. **G**, **H** Levels of interleukin-6 (IL-6) in WAT and BAT. Scale bar = 100 μm. *P < 0.05, **P < 0.01, *n* = 6

### APS inhibited sympathetic activation and IL-6 in HF-induced cachexia

Subsequently, this experiment investigated the mechanisms underlying the APS treatment-induced alterations. Recent studies have shown that adipose tissue metabolism is severely affected by excessive sympathetic activation and inflammation [25–27]. In the present study, both NE and IL-6 were evidently elevated in WAT and BAT of rats with HF-induced cachexia (*P* < 0.01; Fig. 6E-H); moreover, the expression levels of β3 AR and the sympathetic marker Th also increased (*P* < 0.01; Fig. 6A-D) [28–30]. These abnormal levels could be decreased by administration of APS (*P* < 0.01, *P* < 0.05). Thus, the fat wasting in HF-induced cachexia might be due to inflammation and hyperactivation of the sympathetic nervous system, which is improved by APS.

## Discussion

Currently, although a combination of pharmacological agents, including angiotensin-converting enzyme inhibitors and nutritional support are advised to treat cardiac cachexia, there are currently no clear approved treatment options to improve cardiac cachexia through the underlying pathogenesis. Thus, it is valuable and essential to find new drugs for the treatment of cardiac cachexia.

Adipose tissue is crucial for energy metabolism, but excessive adipose wasting in cardiac cachexia in HF has received less attention. A previous study found that the WAT was significantly smaller in HF rats than in normal rats, although there was no significance in body weight; moreover, adipocyte sizes were smaller, with increased small lipid droplets [31]. These results were consistent with studies in this experiment, which have demonstrated that both total fat mass and the total fat-to-body weight ratio were decreased compared with non-HF rats. Meanwhile, both lipid accumulation and adipocyte size in white and brown adipocytes were decreased compared with non-HF rats. WAT is an important organ to maintain the balance of lipid storage and utility. Adipose tissue insufficiency and dysfunction occur in cachexia. Increased lipolysis results in insufficient lipid storage, leading to dyslipidaemia and ectopic fat deposition in peripheral tissues [32]. As shown in the present study, HF-induced cardiac cachexia resulted in elevated blood levels of TG and FFA as well as excessive fat accumulation in the liver. This phenomenon was consistent with previous studies that reported that excessive lipolysis caused by hypermetabolism accelerated the development of fatty liver and increased blood levels of FFAs in cachexia caused by cancer and burn injury [25, 33].

Fat droplets can account for 95% of the total volume of white adipocytes and are mainly composed of TGs. TGs stored within lipid droplets are hydrolysed into fatty acids and released as fuel in peripheral tissues according to metabolic demand. Under lipolysis stimulation, lipid droplet enzymes such as perilipin-1 and HSL on the surface of lipid droplets are phosphorylated by PKA, which promotes ATGL to flow to the surface of lipid droplets and hydrolyzes the first fatty acid from the TGs. Phosphorylated HSL binds perilipin-1,which converts the resulting diacylglycerol into monoacylglycerol, yielding another fatty acid and a glycerol molecule [34, 35]. Part of the FFAs released by lipolysis are exported into the mitochondrial matrix by CPT-1 to undergo β-oxidation [35]. The key enzymes of lipolysis, ATGL and HSL, play a major role in cachexia progression. In patients with cachexia, HSL and ATGL activities are increased [36]. Knockout of any of these lipases prevents cancer-induced adipose tissue loss [36]. Perilipin-1 has been shown to play a central role in regulating the hydrolysis of triglyceride molecules [37]. Adipocytes lacking perilipin-1 fail to translocate HSL into lipid droplets despite activation of the cAMP-PKA signalling pathway [38, 39]. The present study demonstrated that lipolysis activities and FFA β-oxidation were increased in rats with hypertension-induced HF, as characterized by the upregulation of HSL and perilipin-1 as well as the rate-limiting enzyme CPT-1. The above results were consistent with previous findings found in cachexia models induced by abdominal aortic constriction-induced HF and C26 tumours, which demonstrated that activated lipolysis was characterized by elevated expression of HSL and ATGL [40, 41].

Beiging of WAT is usually thought to be a favourable treatment for obesity by burning excessive fat [42]. However, this process may not always be beneficial. Recent studies have discovered beiging of WAT in the development of hypermetabolic diseases such as burns and cancer, and it was thought to be a driver for fat wasting [43–45]. Recently, there have been limited studies reporting the beiging of WAT in HF. Maria et al. [31] found that WAT in EF mice exhibited a brown adipocyte-like phenotype and increased beiging markers, indicating increased beiging of WAT. Another study found high expression of UCP-1 in the BAT of mice with constricted left coronary arteries, suggesting functional overactivation of BAT [46]. These results were consistent in the WAT of HF rats induced by abdominal aortic constriction, which exhibited elevated UCP-1 and PGC1-α mRNA levels [41]. Similarly, the present study also found increased markers of beiging of WAT, such as *Cd137* mRNA and *Zic-1* mRNA, as well as expression of UCP-1 in HF rats induced by salt-sensitive hypertension. This result suggested that both accelerated lipolysis and elevated beiging of WAT are important mechanisms of WAT wasting in HF.

WAT serves mainly as an energy storage organ, while BAT utilizes stored energy to generate heat during thermogenesis, a highly energy costly process. Catecholamine signalling stimulates PKA-p38MAPK signalling, which enhances heat production by upregulating the PGC-1α/PPAR thermogenesis regulatory pathway and/or mobilizing substrates to fuel thermogenesis [47, 48]. Meanwhile, lipolysis-derived FFAs from the extracellular pool are transported into the mitochondria of brown adipocytes via FFA binding and transport proteins such as CD36 and FATP-1 as well as the CPT system [49–51] and serve as substrates for mitochondrial thermogenesis. In this study, it was found that the activated PKA-p38 MAPK signalling was accompanied by regulated PGC-1α, PPAR-γ and UCP-1 in HF rats induced by salt-sensitive hypertension. Additionally, there was elevated FFA utilization since the mRNA levels of FFA binding and transporters such as CD36, FATP-1 and CPT-1 were significantly increased. These results indicated that thermogenesis capability in brown adipocytes is increased compared with rats without HF. A study performed in mice with colorectal cancer cachexia revealed that key regulators of lipid accumulation and fatty acid β-oxidation were upregulated, including expression of UCP1, suggesting active BAT [52]. In severe HF patients, BAT was found to be activated by the use of [18 F]-fluorodeoxyglucose (FDG)-positron emission tomography [53]. These results supported the findings in these salt-sensitive hypertension-induced cardiac cachexia rat models.

Sympathetic nervous system activation and inflammation are the main two upstream molecular mechanisms participating in excessive lipolysis and thermogenesis of adipose tissue in cachexia [37]. After evaluating the plasma NE levels, it was found that the sympathetic nerves in patients with HF were mostly in an overactive state [54]. Early studies have observed that patients with chronic HF who developed cardiac cachexia showed elevated levels of catecholamines [55], and that sympathetic hyperactivation predicted weight loss in HF patients [56]. In severe burn injury and cancer, overactivated sympathetic nerves increase neuronal catecholamine synthesis and secretion, activate β3 AR of adipocytes, and induce WAT browning, leading to a hypermetabolic response [29, 44, 45]. Additionally, HF is characterized by inflammatory activation. The role of inflammatory cytokines in adipose tissue metabolism cannot be ignored. Prolonged activation of IL-6 signaling induces excessive consumption of adipose tissue and increases energy expenditure in cancer by accelerating lipolysis and adipose tissue browning [43, 57]. All the above results suggested that inhibiting sympathetic nerves and inflammation may ameliorate fat wasting in cachexia. Using cardiac cachexia rats induced by abdominal aortic constriction, a significant increase in serum NE and IL-6 levels have been observed, which may be one reason for the BAT [41]. Interestingly, it was also observed in this study that the expression of β3 AR, sympathetic nerve fibre distribution and the levels of NE and IL-6 in both WAT and BAT were increased in these salt-sensitive hypertension-induced cardiac cachexia rat models. The therapeutic role of APS in metabolic diseases such as obesity, fatty liver and diabetes has been widely studied. The anti-inflammatory, hypoglycaemic, hypolipidaemic, and insulin resistance-improving functions of APS have become the focus of therapeutic attention. However, the effect of APS on adipose tissue wasting in cardiac cachexia has not been implicated in previous studies.

**Fig. 7:**
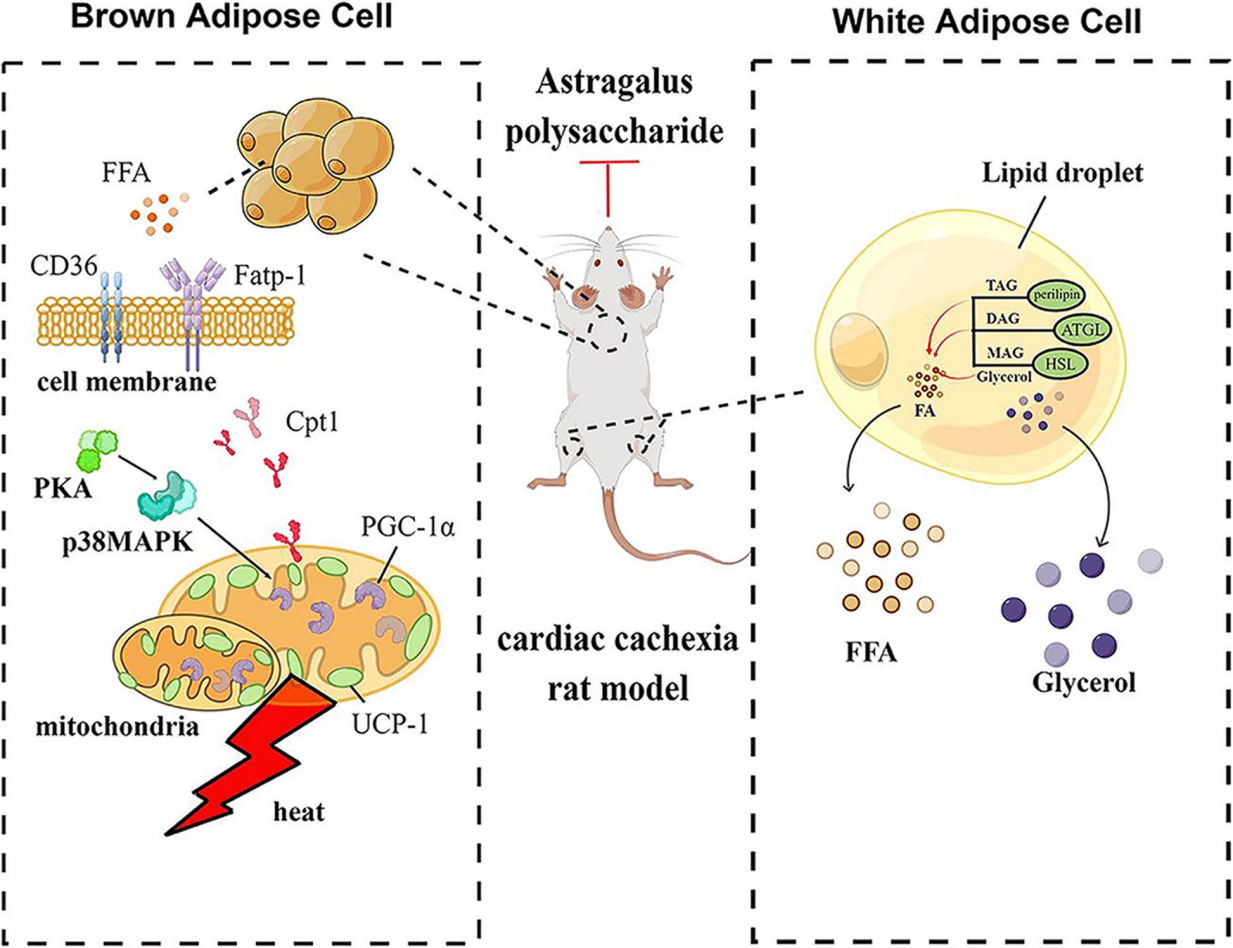
Mechanisms by which APS prevents adipose consumption in HF-induced cardiac cachexia. Elevated lipolysis and browning of white adipocytes in WAT as well as thermogenesis in BAT contribute to adipose consumption. Treatment with APS alleviated adipose consumption by inhibiting lipolysis and browning of white adipocytes in WAT and decreasing thermogenesis in BAT. This figure was drawn in Figdraw

A previous study reported that APS decreased blood levels of FFAs by increasing FAT/CD36 to promote the synthesis of TG [58]. Moreover, APS alleviated lipid droplet deposition in the liver. Consistently, in this study, it was also found that APS treatment decreased the circulation contents of FFA and TG and decreased ectopic lipid deposition in the liver. Additionally, adipocytes stained with HE and Oil red O exhibited markedly larger lipid droplets, suggesting that APS treatment favours lipid storage in adipocytes. The results of this experiment are consistent with previous studies, which reported that APS significantly facilitated lipid accumulation in C3H10T 1/2 cells [59]. Interestingly, we also found that APS inhibited lipolysis and browning of white adipocytes in WAT, as shown by the expression of lipid droplet enzymes, including HSL and perilipin, and beige adipocyte markers, including UPC-1, Cd137 and Zic-1, were suppressed after administration of APS. Additionally, treatment with APS decreased thermogenesis, as reflected by decreased expression of UCP-1, PPAR-γ and PGC-1α, and reduced FFA β-oxidation in mitochondria, as reflected by decreased expression of *Cd36*, *Fatp-1* and *Cpt1* in BAT. These effects were related to inhibition of the PKA-p38 MAPK signalling pathway. As shown in Fig. 7, the results suggested that APS prevented adipose expenditure by alleviating lipolysis in white adipocytes and thermogenesis in beige/brown adipocytes under conditions of cardiac cachexia. A previous study showed the anti-inflammatory effect of APS by downregulating proinflammatory cytokines [60]. Through further analysis in this study, it was found that APS could downregulate the expression of IL-6 in adipose tissue. Additionally, APS inhibited sympathetic activity and reduced sympathetic density in adipose tissue. Therefore, these pronounced anti-inflammatory effects and sympathetic suppression associated with APS may play a major role in protecting excessive adipose expenditure in cardiac cachexia.

### Comparisons with other studies and what does the current work add to the existing knowledge

Excessive fat consumption has previously been shown to be strongly associated with the degree of cardiac cachexia. However, there is still a lack of an effective drug for cachexia. This study provides the first evidence that APS prevents excessive fat consumption by inhibiting lipolysis and adipose browning in WAT and decreasing BAT thermogenesis. These effects may be related to suppressed sympathetic activity and inflammation. This study provides a potential drug to treat HF-induced cardiac cachexia.

### Study strengths and limitations

The strength of this study is that it has provided evidence that Astragalus APS could alleviate adipose consumption by inhibiting lipolysis and browning of white adipocytes in WAT and decreasing thermogenesis in BAT in a rat model of HF-induced cardiac cachexia, providing a new idea for the clinical development and treatment of new drugs for cardiogenic cachexia. This study also had some limitations. Although pair feeding was used during this study to minimize the effect of food intake, differences in food intake can affect body weight and fat mass. Moreover, the activity factors of the rats were not recorded, which is also a limitation of this study. Additionally, this study ignored the effect of a high-salt diet on adipose tissue metabolism. A study performed by Vanessa et al [61] showed that a high-salt diet had little impact on visceral fat mass and adipocyte size. However, Cui et al. [62] found that the intake of a high concentration of sodium chloride (96% NS + 4% NaCl) can inhibit fat deposition and downregulate the expression of some genes related to lipogenesis. These findings did not interfere with the interpretation of the effects of APS in this study. Finally, this study only focuses on the anti-inflammatory and sympathetic inhibition effects of APS, which cannot fully explain the diversity of APS pharmacological effects. The protective mechanism of APS in cardiac cachexia needs to be further studied.

## Conclusion

In conclusion, the present study demonstrated that the salt-sensitive hypertension-induced cardiac cachexia rat model showed significant adipocyte atrophy and FFA efflux, elevated lipolysis and browning of white adipocytes in WAT and thermogenesis in BAT. Inflammation and an overactivated sympathetic nervous system may be one of the most upstream mechanisms of fat wasting in HF-induced cardiac cachexia. Treatment with APS inhibited lipolysis and browning of white adipocytes in WAT and decreased thermogenesis in BAT; moreover, these effects of APS may be related to suppressed sympathetic activity and inflammation. These results suggested that APS reversed HF-induced cardiac cachexia by preventing excessive adipose expenditure, partly by reducing inflammation and downregulating activation of the sympathetic nervous system. This study provides ideas for the development of new drugs for cachexia, which may help improve the quality of life and prolong life of patients with end-stage HF. Further studies should focus on illustrating the pharmacological action of APS on adipose metabolism in the context of cachexia.

## Supplementary Information

**Additional file 1.**

**Additional file 2.**

## Data Availability

The datasets used and/or analyzed during the current study are available from the corresponding author on reasonable request.

## Abbreviations

AC: adenylate cyclase
APS: Astragalus polysaccharide
ATGL: Adipose triglyceride lipase
BAT: brown adipose tissue
β-ARs: β-adrenoreceptors
CPT1: Carnitine Palmitoyltransferase
EF: Ejection Fraction
FATP-1: Fatty Acid Transport Protein-1
FFAs: Free Fatty Acids
FS: Fractional Shortening
GAPDH: Glyceraldehyde3-Phosphate Dehydrogenase
HF: Heart Failure
HSL: Hormone-Sensitive Lipase
IL-6: Interleukin 6
NE: Norepinephrine
PGC-1α: Peroxisome Proliferator-Activated Receptor γ Coactivator 1α
p38 MAPK: p38 mitogen-activated protein kinase
PPAR-γ: Peroxisome Proliferator-Activated Receptor Gamma
PKA: Protein Kinase A
TG: Triglycerides
Th: Tyrosine Hydroxylase
UCP-1: Uncoupling Protein-1
WAT: White Adipose Tissue
